# Cell survival and differentiation with nanocrystalline glass-like carbon using substantia nigra dopaminergic cells derived from transgenic mouse embryos

**DOI:** 10.1371/journal.pone.0173978

**Published:** 2017-03-23

**Authors:** Noela Rodriguez-Losada, Pablo Romero, Guillermo Estivill-Torrús, Roberto Guzmán de Villoria, Jose A. Aguirre

**Affiliations:** 1 Department of Human Physiology, Faculty of Medicine, University of Malaga and Biomedicine Biomedical Research Institute of Malaga (IBIMA), Campus de Teatinos, Boulevard Louis Pasteur, Malaga, Spain; 2 IMDEA Material Institute, C/Eric Kandel 2, Getafe, Madrid, Spain; 3 Unidad de Clínica de Neurociencia, Biomedical Research Institute of Malaga (IBIMA), Regional University Hospital Malaga, Av. de Carlos Haya, s/n, Málaga, Spain; 4 FIDAMC. Foundation for the Research, Development and Application of Composite Materials Avda. Rita Levi-Montalcini 29, Getafe, Madrid, Spain; Kyoto Daigaku, JAPAN

## Abstract

Regenerative medicine requires, in many cases, physical supports to facilitate appropriate cellular architecture, cell polarization and the improvement of the correct differentiation processes of embryonic stem cells, induced pluripotent cells or adult cells. Because the interest in carbon nanomaterials has grown within the last decade in light of a wide variety of applications, the aim of this study was to test and evaluate the suitability and cytocompatibility of a particular nanometer-thin nanocrystalline glass-like carbon film (NGLC) composed of curved graphene flakes joined by an amorphous carbon matrix. This material is a disordered structure with high transparency and electrical conductivity. For this purpose, we used a cell line (SN4741) from substantia nigra dopaminergic cells derived from transgenic mouse embryos. Cells were cultured either in a powder of increasing concentrations of NGLC microflakes (82±37μm) in the medium or on top of nanometer-thin films bathed in the same culture medium. The metabolism activity of SN4741 cells in presence of NGLC was assessed using methylthiazolyldiphenyl-tetrazolium (MTT) and apoptosis/necrosis flow cytometry assay respectively. Growth and proliferation as well as senescence were demonstrated by western blot (WB) of proliferating cell nuclear antigen (PCNA), monoclonal phosphorylate Histone 3 (serine 10) (PH3) and SMP30 marker. Specific dopaminergic differentiation was confirmed by the WB analysis of tyrosine hydroxylase (TH). Cell maturation and neural capability were characterized using specific markers (SYP: synaptophysin and GIRK2: G-protein-regulated inward-rectifier potassium channel 2 protein) via immunofluorescence and coexistence measurements. The results demonstrated cell positive biocompatibility with different concentrations of NGLC. The cells underwent a process of adaptation of SN4741 cells to NGLC where their metabolism decreases. This process is related to a decrease of PH3 expression and significant increase SMP30 related to senescence processes. After 7 days, the cells increased the expression of TH and PCNA that is related to processes of DNA replication.

On the other hand, cells cultured on top of the film showed axonal-like alignment, edge orientation, and network-like images after 7 days. Neuronal capability was demonstrated to a certain extent through the analysis of significant coexistence between SYP and GIRK2. Furthermore, we found a direct relationship between the thickness of the films and cell maturation. Although these findings share certain similarities to our previous findings with graphene oxide and its derivatives, this particular nanomaterial possesses the advantages of high conductivity and transparency. In conclusion, NGLC could represent a new platform for biomedical applications, such as for use in neural tissue engineering and biocompatible devices.

## Introduction

Regenerative medicine requires, in many cases, physical supports to facilitate appropriate cellular architecture, cell polarization and the improvement of the correct differentiation processes of embryonic stem cells, induced pluripotent cells or adult cells. Interest in carbon nanomaterials with high transparency and electrical conductivity has grown within the last decade in light of a wide variety of applications, including their use in biocompatible sensors, diagnostic devices and bioelectronic implants [1]. In the case of neuronal differentiation, eligible materials as scaffolds also possess special characteristics, such as controllable surface morphology, flexibility (controlled thickness), hydrophilic nature, electric conductivity and, in some cases, transparency (depending on the thickness) to follow the growth of cultures [2][3][4][5]. Some carbon crystalline structures, such as graphene, nanotubes, nanofibers and fullerenes, and disordered structures, such as diamond-like carbon, glass-like carbon, and amorphous carbon, are now being considered as possible scaffoldings, and therefore, studies of their biocompatibilities have begun to be reported [3][6] [7][8] [9][10] [11]. Among the crystalline structures, graphene [2] and, in particular, graphene oxide [4][12] and its derivatives have provided remarkable results for cell proliferation and neuronal differentiation, although the applicability has been hampered by evidence of nanotoxic effects on different cell types [6]. Among the second group, disordered structures, diamond-like carbon has been proposed as biocompatible and bioactive surface coatings that can promote and stabilize cell attachment [10], promotes the formation of functional neuronal networks [11] and can be use as a tailorable and tunable substrate to study neural cell biology in vitro and in vivo [13].

Therefore, new carbon-based biomaterials that offer biocompatible and mechanically stable platforms for cell growth require exploration. Recently, particular nanometer-thin nanocrystalline glass-like carbon films (NGLC) composed of curved graphene flakes joined by an amorphous carbon matrix have undergone controlled synthesis by atmospheric pressure chemical vapor deposition on copper foils. This material replicates the structure of the long-term widely used glass-like carbons [14]. Thus, NGLC is related to graphene because it constitutes a building-block of its general microstructure but exhibits an overall amorphous and disordered nature. NGLC can also be produced in the form of thin films, which possess similar physicochemical, electric and optical properties of graphene, such as high transparency (5 nm-tick films: 86% and 20 nm-tick-film: 59%) and moderate electrical resistance (5 nm-tick films: 7.8 kΩ/sq and 20 nm-tick-film: 4.2 kΩ/sq). The control of the optical and electrical conductivities of these nanostructured carbon thin films based on the film thickness has been demonstrated [15].

In this study, we evaluate the capacity of NGLC in the form of thin films (5–80 nm) and powder suspended in the cell culture medium for biocompatibility, which would promote normal cell growth, cell differentiation and cell maturation, for which, in the case of this new carbon-based biomaterial, no data have been reported to date. In this work, we utilized the progenitor cell line SN4741 from substantia nigra dopaminergic cells derived from transgenic mouse embryos [16]. Because SN4741 cells are undifferentiated cloned cells with a constant proliferative speed of 36 hours when grown under standard conditions and a well-characterized fibroblast-like morphology, any observable change in their features would depend exclusively to the direct effect of the NGLC microparticles. Our results demonstrate NGLC as a biocompatible material in our model and highlight the potential of NGLC to be used in many biomedical applications requiring a substrate for neural repairing/support. In this sense, given the reported cell replacement therapies for damaged dopaminergic cells in Parkinson’s disease [17] our findings made NGLC a very capable substrate for neural cell growth to be considered for neuronal engraftment and tissue engineering.

## Materials and methods

### Synthesis of NGLC films and microflakes

In this study, we used recently produced NGLC thin films [15] derived through controlled synthesis by atmospheric pressure chemical vapor deposition (CVD) on copper foils from which we also produced NGLC powder (microflakes). The CVD process consists of the thermal decomposition of a carbon source (C_2_H_4_ at 850°C) on top of a copper foil (22×60 mm^2^) in a controlled atmosphere including Ar and H_2_. We used three different gas compositions to obtain different carbon films with different thicknesses (values in sccm, Ar/H_2_/C_2_H_4_ = 500/20/20, 300/120/120 and 0/70/70 corresponding to ~ 5 nm, 20 nm and 80 nm thick films).

We transferred the synthesized carbon films to drop-casted polymer poly (methyl methacrylate) (PMMA) diluted in anisole (495PMMA Resists, Microchem). First, we homogeneously distributed ~ 0.5 ml of PMMA on top of the carbon film/copper using a plastic disposable pipette. We let it air dry (12 h) and repeated the process two more times. This process resulted in a PMMA film thickness of 20–40 μm. Then, the PMMA/carbon film/copper system was placed on a copper etchant bath to dissolve the copper foil (Alpha Aesar standard). PMMA was placed at the bottom and totally submerged in the etchant bath. We replaced the entire whole copper etchant for a new fresh one after two hours and let it etch for 24 hours. The resulting carbon film/PMMA film was cleaned by removing the copper etchant with clean deionized water, and we replaced the water 5 times. Next, it was cleaned in the deionized water bath for 24 hours. After thoroughly cleaning again with deionized water, the sample (with the carbon film facing upwards and the PMMA film backwards) was ‘fished’ with a clean transparent film (PVC A4 180 μm CE011880E) and was let dry for 24 hours. Finally, we cut the sample in slices (8x8 mm^2^ approx.) with a razor blade. To evaluate the effect of the PMMA and the copper roughness, we prepared two baseline samples. First, we prepared a PMMA/copper sample following the transfer process described above using a raw copper sample that had not been introduced in the CVD reactor (non-carbon film process). We also prepared a copper sample following the same CVD synthesis process to grow an NGLC film but with a different gas composition (Ar/H_2_/C_2_H_4_ = 1000/20/0 sccm) (thermal-treated copper film). As we did not flush any hydrocarbon during the CVD process, this ‘thermal-treated copper foil’ sample did not have an NGLC on top of it, but it mimicked the copper roughness produced during the CVD process.

The production of NGLC powder was carried out by a first CVD on a rolled copper foil (10x30 cm^2^) using a gas concentration of Ar/H_2_/C_2_H_4_ = 100/20/20 for 60 minutes. This CVD resulted in the deposition of a ~300-nm-thick carbon film. To produce a dispersion of NGLC powder, we introduced 300-nm-thick NGLC film deposited on copper to an ethanol bath, and we detached the film and broke it into flakes via high-energy tip sonication (1 minute, 40% of amplitude and 1 second pulses, Vibra-Cell 500 W). We analyzed the NGLC films by transmission electron microscopy (JEOL JEM 3000F) and both the films and powder by scanning electron microscopy (EVO MA15, Zeiss) and Raman spectroscopy using a Nd:YAG green laser (532 nm, Jasco, NRS-5100), and we compared them to a commercial graphene sheet deposited on a silicon wafer (Graphenea, Spain). Surface topography of the NGLC film/PMMA composite was characterized by atomic force microscopy (AFM) at ambient conditions using a Park XE150. The images were acquired in non-contact mode using a non-contact cantilever (PPP-NCHR, Park System) with a tip set point of approximately ~30–40 nm and amplitudes between ~25–45 nm with a scan rate of 0.50 Hz. The obtained images (512x512 pixels and areas of 10 μm^2^) were processed and analyzed using XEI software (version 1.7.1).

### Microflakes

We tested in this study the effects of five different NGLC powder (microflakes) concentrations (1, 5, 10, 20 and 50 μg/ml). In a separate experiment, we used NGLC films with three different thicknesses (5-, 20- and 80-nm-thick films) and transparency properties to allow the cells to grow on top of the film. Prior to starting the experiments, the NGLC material underwent 15 min of UV irradiation at 185 nm. The material was confirmed to be microplasma-free (Cell Culture Contamination Detection Kit C7028, Thermo Fisher Scientific, USA). Next, the material was confirmed to be free of any biological contamination and to provide a reversible wettability hydrophobic to hydrophilic transition that rendered the surface of the NGLC highly biocompatible and facilitated adhesion, as has been recently published in relation to the same glass-like carbon [18].

### Cells and culture conditions using NGLC microflakes and films

The SN4741 cell line from substantia nigra dopaminergic cells derived from transgenic mouse embryos [16] provided by Prof. E. Arenas (Karolinska Institute, Stockholm, Sweden) was used in this study. The cells were grown in normal culture conditions with DMEM supplemented with 10% FCS, glucose (0.6%), penicillin-streptomycin (50 U/ml and 50 μg/ml respectively) and L-glutamine (2 mM) in a 5% CO_2_ humidified atmosphere, as previously described [19]. Cells were thawed and placed in culture medium; after 5 passages the cell growth was synchronized. A growth curve was used to establish the rate of doubling time calibration [20]. The cells were exposed to NGLC microflakes (size 5-10 μm^2^) and cultured in five concentrations (1, 5, 10, 20 and 50 μg/ml) for 24 h, 3 days or 7 days. In all cases, a stock microflakes solution (2 g/l) in essential medium without phenolphthalein was used. No addition of NGCL was used as control and similarly tested. In a separate experiment, we used NGLC films with three different thicknesses (5-, 20- and 80-nm-thick films) and transparency properties to allow the cells to grow (7 days) on top of the film to evaluate the ability of sustaining mature-DA cells to scaffold. Morphological evolution of the cells was assessed by using an inverted TE2000-U microscope joined to a Nikon DS5MC camera (Izasa, Spain) and a Zeiss LSM700 confocal microscope (Zeiss, Germany). Photographs were taken after 24 hours, 3 and 7 days of treatment, and the morphological changes were analyzed by comparing them with SN4741 cells cultured in the same conditions but without NGLC.

The analysis of morphological changes was performed by Huang-Threshold-Auto software [21][22] and the Analysis of Skeletonize 2D/3D used the method of shortest branch [23]. To measure the length of the branches we used the Measurement Microscope Tools Pluging (http://fiji.sc/Microscope_Measurement_Tools). The Canny Edge Detector Pluging [24] was used to find the initial segments of the most marked borders/edge. All plugins were obtained from GitHub source repository (https://imagej.nih.gov/ij/plugins).

### Assessment of NGLC microflakes capacity on metabolism activity

Cellular metabolism activation was tested using the MTT reduction assay [20][25], after exposing SN4741 cells to microflakes in time and concentrations above mentioned. The experiments were conducted in 96-well plates (Corning) using 2500 cells per well cultured in DMEM supplemented with 10% FCS penicillin streptomycin (1000 U/ml) and incubated at 37°C in humidified 5% CO2. The analysis of methylthiazolyldiphenyl-tetrazolium (MTT) was performed following the manufacture’s recommendations (Roche, MTT kit assay) using a positive control with 10% Triton X-100 treatment (15 minutes) and a negative control with neither Triton X-100 nor NGLC. The reaction was read using a BioTek (Izasa, Spain) luminescence reader at a test wavelength of 570 nm with a reference wavelength of 630 nm. The analysis of viability was considered as a rate of the viability: 100 - (sample-positive control with Triton X-100 (10%)) x 100% as previously described in Roche’s protocol for the MTT metabolic assay considering that MTT tetrazolium salt may be reduced not only in the mitochondria but also within the cytoplasm, on the surface of cell, endosome or lysosome membranes, or even in the extracellular environment [25].

### Apoptosis/Necrosis assay

To discriminate between live (viable) and dead cells (cells with damaged membranes, including apoptotic and necrotic signs) a LIVE/DEAD® Viability/Cytotoxicity Assay Kit (Invitrogen, CA, USA) was used. SN4741 cells cultured in 6 wells (150000 cells per well) with addition of NGLC microflakes at the same above cited concentrations (1–50 μg/ml). After 7 days of culture, cells were processed following the manufacturer recommendations. The stained cells were counted by flow cytometry using BD Accuri^TM^ (BD bioscience, San Jose, CA, USA) software for analysis. Also the analysis was corroborated using immunofluorescence (inverted microscopy TE2000-U joined to a camera Nikon DS5MC) in vivo of cells cultured with NGLC microflakes.

### Western-blot

SN4741 cells treated with NGLC for 24h and 7 days as well as controls were grown in 60-mm well plates under various experimental conditions, rinsed twice with PBS 1x and lysed using 100 μl of RIPA buffer containing proteases inhibitors (1% NP-40; 0.5% Na deoxycholate; 0.1% SDS; PMSF 100 μg/ml; aprotinin 30 μl/ml; Na orthovanadate 1 mM). The lysate was scraped off and transferred to a microcentrifuge tube, passed through a 21-gauge needle, and centrifuged at 16000 rpm for 20 min at 4°C. The supernatant was used as the total cell lysed. The lysate (20 μg of cell) was heated at 95°C for 5 minutes in Laemmli buffer 1x and then analyzed by sodium dodecyl sulphate-polyacrylamide gel electrophoresis (10%) at 15 V/cm, 1 h. The protein was transferred to a polyvinylidene difluoride membrane for immunoblotting and was incubated for 4 hours at RT in the blocking buffer containing 5% non-fat dry milk in TBS buffer (0.1% Tween 20 in 0.1% TBS) [16]. Protein detection was performed using the primary antibodies against polyclonal anti-rabbit proliferating cell nuclear antigen (PCNA) (1:5000, Abcam), polyclonal anti-rabbit anti-tyrosine hydroxylase (TH, Sigma-Aldrich) (1:1000), mouse monoclonal phosphorylate Histone 3 antibody (serine 10) (PH3, Abcam) and mouse monoclonal SMP30 antibody (G-10; 1:500 Santa Cruz Biotechnology, USA). The primary antibodies were incubated at 4°C overnight in a shaker with horseradish peroxidase-conjugated antibody (1:10000) diluted in blocking buffer. Signals were detected with horseradish peroxidase ECL systems (Thermo Fisher, Rockford USA). Images and quantification of bands were analyzed using Fiji-ImageJ software.

### Evaluation of the effect of NGLC films on neuronal capability

SN4741 cells were cultures on top of the NGLC films (5, 20 and 80 nm thickness) for a months, and controls were fixed with 4% paraformaldehyde (w/v) in 0.1 M PBS, pH 7.4, during 20 min, rinsed three times with PBS and permeabilized with 0.1% Triton X-100 in PBS. The cells were blocked with 5% normal goat serum (NGS, Invitrogen) in PBS in 3% BSA (Invitrogen). The following antibodies were used: anti-synaptophysin (SYP, clone SY38, Chemicon Millipore, Massachusetts, USA) and goat anti-GIRK2 (G-protein-regulated inward-rectifier potassium channel 2 protein) (Abcam, Cambridge, UK). Primary markers antibodies were used at 1:100 in PBS with 3% BSA overnight at 4°C. After incubation, cells were washed in PBS 0.1% Tween-20 for 1 hr at room temperature (RT). Secondary antibodies (Alexa Fluor 488 goat anti-mouse; Alexa Fluor 555 goat anti-rabbit) were incubated with cells for 2 hours at a concentration 1:500, RT, in PBS with 3% BSA. Cells were washed for 1 h in PBS in 0.1% Tween-20, and finally, the cells were stained with DAPI (nuclei marker, Sigma) at 1:200 for 15 minutes at RT. The analysis of the colocalization coefficient for fluorescence microscopy to quantify protein interactions was performed using Pearson's correlation coefficient, as previously described [26], using the image processing package Fiji-ImageJ with the Colocalization Threshold plugin. The counter of total cells (nuclei counting) in each film was performed using Counter Particle Plugin and Automatic cell counting Plugin [27]. In vivo analysis of the SN4741 cell adherence on NGLC films was performed on cultures of 200000 cells (high concentration) in 24 wells (Nunc) using Hoescht 33342 (1 μg/ml) as marker. Samples were incubated for 15 min and then visualized using in an inverted fluorescence microscopy TE2000-U joined to a camera Nikon DS5MC. Samples were quantified using ImageJ-software.

### Statistical analysis

Statistically significant differences between datasets were assessed by Student’s tests (after validation of variance homogeneity by Levene’s test) for parametric data and by one-way ANOVA with Fisher’s post hoc test for nonparametric data. A *p*-value < 0.05 was considered statistically significant. The data were analyzed using the GraphPad Prism data analysis program (GraphPad Software, San Diego, CA). The data are expressed as the mean±standard error of the mean (SEM). The same experiment was repeated at least three times.

## Results

### NGLC microflakes and films

**Fig 1:**
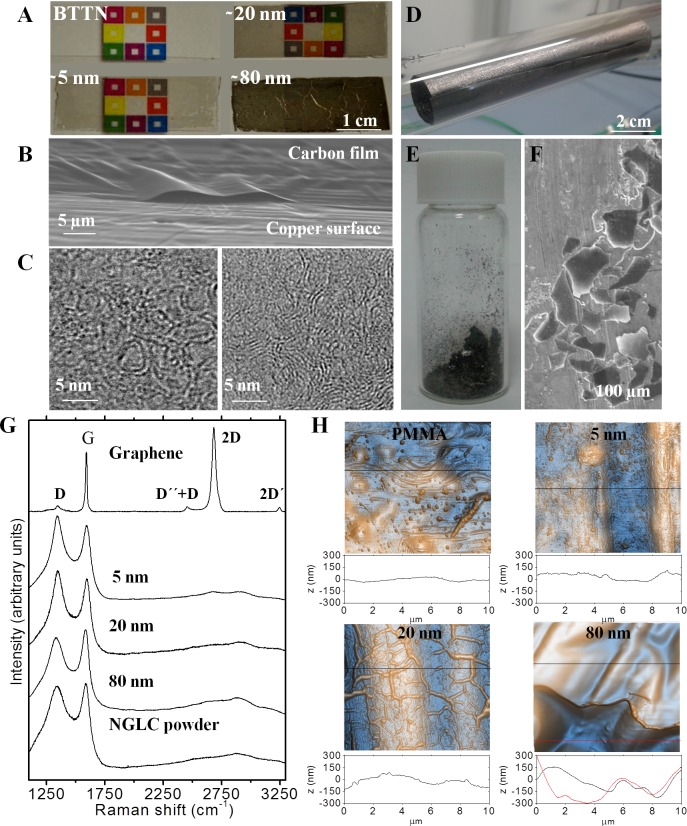
Images and characterization of nanocrystalline glass-like carbon (NGLC) films using different microscopy techniques (optical, electron microscopy and atomic force microscopy (AFM)). NGLC films with different thicknesses (~5, ~20 and ~80 nm) and baseline thermal treatment non-carbon film (BTTN) used in the experiments showing different degrees of transparency (**A**). The thinnest sample (~5 nm) shows the high transparency (86%) and moderately high electrical conductivity (sheet resistance: 7.8 kΩ/sq). They were obtained on a copper surface by carefully controlling the gas flow ratios used in the CVD procedure (see Material and Methods) (**B**). These nanostructured carbon thin films are composed of few-layer and curved graphene fragments of ~3 nm in average size joined by an amorphous carbon matrix (**C**), which replicates the structure of widely used glass-like carbons. Carbon-coated copper after CVD (**D**) and flakes of 82±37 μm length and approximately 300 nm thicknesses (**E**, **F**). Raman spectroscopy of NGLC films with different thicknesses (~5, ~20 and ~80 nm), microflakes and graphene, showing the broad spectra of amorphous carbons compared to highly crystalline graphene (**G**). Surface roughness of PMMA/Carbon film composites measured by AFM, showing increasing roughness on those with a 20- and 80-nm-thick carbon films (**H**). See also Romero et al., 2016 [15] (Doi:10.1016/j.cej.2016.04.005).

For this work we used 5-, 20- and 80-nm-film thick NGLC on top of PMMA supports and NGLC powder (microflakes) in SN4741 cell cultures. As was extensively described in our recent publication [15], we were able to define the thickness of these films, and thus their optical and electrical properties, by carefully controlling the gas flow ratios used in the CVD procedure (Fig 1A and 1B). The thinnest samples (~5 nm) showed the high transparency (86%) (Fig 1A) and a moderately high electrical resistance (7.8 Ω/sq). These nanostructured carbon thin films are composed of few-layer and curved graphene fragments of ~3 nm in average size joined by an amorphous carbon matrix (Fig 1C), which replicates the structure of widely used glass-like carbons. Regarding the production of carbon powder by sonication of carbon-coated copper (Fig 1D), we observed that they are composed of microflakes with length of 82±37 μm and approximately 300 nm thickness (Fig 1E and 1F). The crystal structure of our carbon materials was also analyzed by Raman spectroscopy (Fig 1G). The disorder in the microstructure of our carbon films and flakes compared to graphene is evidenced by the appearance of the D peak at *ca*. 1350 cm^-1^, the G band broadening (appearance of the Dʹ peak *ca*. 1620 cm^-1^), and the Dʹʹ peak (*ca*. 1200 cm^–1^). The peaks at *ca*. 2450 cm^-1^ and *ca*. 2950 cm^−1^ are the combination of D, Dʹ, and Dʹʹ bands. Peaks at *ca*. 2700 cm^-1^ and 3250 cm^−1^ are the D and Dʹ overtones respectively, and disappeared in amorphous carbons which is a Raman contribution of Si wafer support on graphene and thin carbon film [28].

Regarding the surface topography of the carbon film samples transferred on PMMA, AFM showed that the roughness increases with increasing NGLC film thickness (Fig 1H). It is known that these wrinkles appear in the carbon film during cooling immediately after the CVD process due to the mismatch between the thermal coefficient of the carbon films and the copper catalyst, and remain once they are transferred to the PMMA. In this study, we tested the effects of five different NGLC microflakes concentrations (1, 5, 10, 20 and 50 μg/ml). In a separate experiment, we used NGLC films with three different thicknesses (5, 20 and 80 nm), and thus transparencies, to allow cells to grow on top of the carbon film. Prior starting the experiments, the material underwent a short (15 min) UV irradiation at 185 nm. The material was confirmed to be microplasma-free, as assessed using a commercial kit used (Thermo Fisher).

### NGLC microflakes effects on SN4741 cells. Analysis of cell morphology and distribution

**Fig 2:**
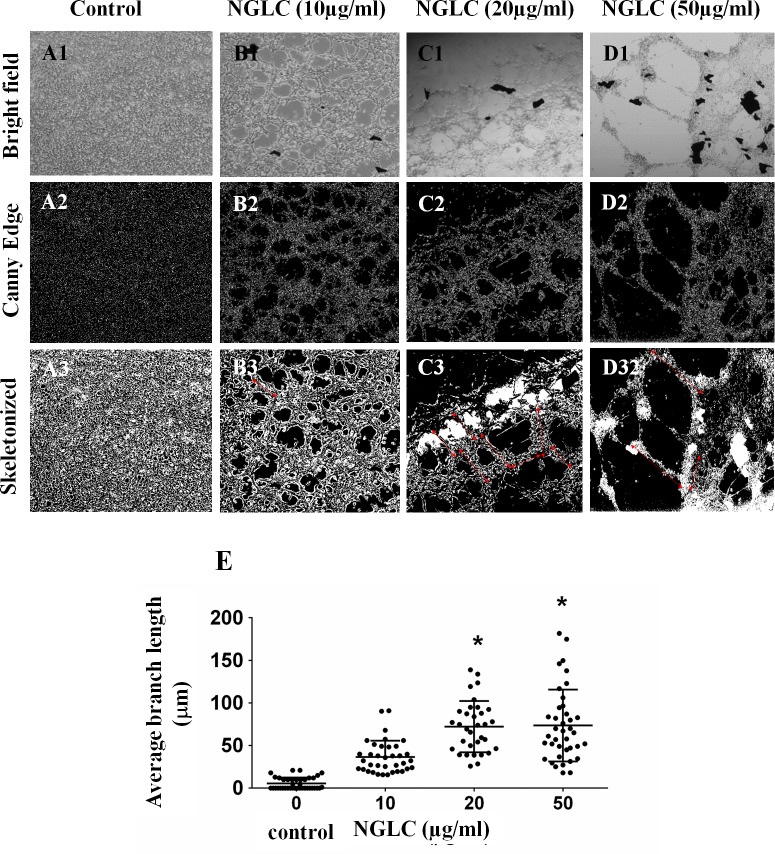
Influence of the concentration of nanocrystalline glass-like carbon (NGLC) microflakes on cell growth. Changes in the cellular architecture in a monolayer SN4741 cells culture (**A-D**) after 7 days of culture with or without (control) NGLC microflakes at three different concentrations (10, 20 and 50 μg/ml). Control is shown in a monolayer culture with high confluence after 7 days (**A1**). An image analysis was performed using Image J/Fiji software and the pluging Canny Edge which inform about the outline structure (**A2**). A cellular architectural analysis was also done by using the plugin Skeletonized 2D/3D (**A3**). Brightfield of the same experiment at NGLC 10 μg/ml (**B1**) showing higher cellular network organization (**B2**) demonstrated by the skeletonized analysis (**B3**). Treatment with NGLC 20 μg/ml (**C1**) shows higher structural changes respect to the control (**C2**) producing greater networks (**C3**: see arrows). Cell culture with NGCL 50 μg/ml (**D1**) shows a cellular network with the greater branches among cell populations (**D2**) as it is indicated by the arrows (**D3**). Quantification of the branches length (**E**) was obtained by analysis of Skeletonized 2D/3D and Microscope Measurement Tools, ImageJ/Fiji software. Magnification = 5X. One-way ANOVA with Fisher’s post hoc test was used for comparison between different groups: *p<0.05 groups vs control.

The changing morphology of the SN4741 cell line was analyzed after 7 days of exposure to NGLC microflakes. Microflakes appeared on the bright field microphotographs as black dots, around which cells grew in organized networks on the culture in a time-dependent manner (Fig 2).

It is shown a cellular monolayer arrangement (Fig 2A1), whose intercellular spaces are barely present after 7 days of culture in a high cell concentration. The analysis of the data of the distance between branches (Fig 2E) shows that control (without treatment) has a mean average branch length of 5.3±1.3 μm (mean±SEM) after measurement of 25 branches (experiment 1: 5.3±2.4 μm (n = 10); experiment 2: 5.3±2.4 (n = 6); experiment 3: 8.0±3.2 (n = 6)). However, we found in the experiment with NGLC 10μg/ml a mean average branch length of 36.6 ±3.2 μm analyzing 36 branches (experiment 1: mean 32.1±2.9 (n = 13); experiment 2: 50.5±6.1 (n = 10); experiment 3: 30.6±5.8 (n = 10)). The sample with NGLC 20 μg /ml has a mean average branch length of 72.2±5.2 μm analyzing 33 branches (experiment 1: 69.4 ± 9.5 (n = 13); experiment 2: mean 75.4±8.0 (n = 13); experiment 3: 71.6±10.5 (n = 7)) where a significant difference was observed respect to the control (p <0.05). In the case of the sample with NGLC 50 μg/ml the mean average branch length was 73.5±6.7 μm analyzing 39 branches (experiment 1: 52.3±6.5 (n = 13); experiment 2: 59.7±9.5 (n = 13); ±13.20 (n = 13)) where a significant difference (p <0.05) was observed respect to control.

### Comparison of cellular metabolic activity and viability

**Fig 3:**
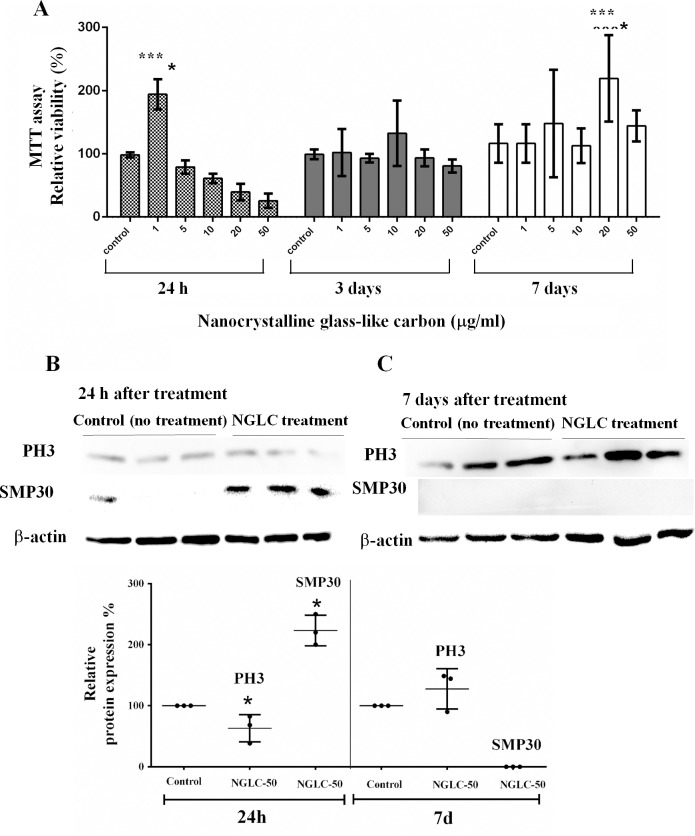
MTT assay for cell metabolism and proliferation. SN4741 were cultured in media containing five different microflakes concentrations (0 as control, 1, 5, 10, 20 and 50 μg/ml) of nanocrystalline glass-like carbon (NGLC) for 24 h, 3 days and 7 days (**A**). Phosphorylate histone 3 (PH3) and SMP30 analysis by SDS-page western blot, shows the analysis at 24 h (**B**) and 7 days (**C**) using NGLC at 50 μg/ml vs control (without NGLC). Quantification of relative PH3 and SMP30 proteins expression (%) showing the normalized data with control sample at 24 hours and 7 days of culture (**D)**. Data are expressed as mean±standard error of the means (SEM, n = 6). One-way ANOVA with Fisher’s post hoc test was used for comparison between different groups; ***p<0.001 groups vs control.

To determine cell metabolic activity the colorimetric MTT metabolic activity assay was used [29]. MTT assay at 24 h, 3 days and 7 days of cell culture with five concentrations of NGLC microflakes (Fig 3) revealed different behavioral patterns.

After 24 hours of culture, a high and significant increase in relative viability (200±24%; p<0.001) was observed with low amounts of NGLC (1 μg/ml), while metabolism decreases as the concentration rises. The lowest metabolic activity is recorded at 24 h with NGLC 50 μg/ml. After three days of culture, the metabolism remained stable respect to the control but, after 7 days, the metabolism enhanced significantly (225±69%; p<0.001) with NGLC 20 μg/ml concentration (Fig 3A). After 24 hours of culture, a high and significant increase in relative metabolism capacity (200±24%; p<0.001) was observed with low amounts of NGLC (1 μg/ml), while metabolism decreases as the concentration rises. In order to interpret the decrease of cell metabolism at 24 h of culture, a study of the PH3 and SMP30 protein expression has been carried out (Fig 3B). Phosphorylation of the highly preserved serine 10 residues of histones is determinant for onset of mitosis (M phase) [30] and thus, the PH3 activation is linked to the split of chromosomes at the beginning of the mitotic cycle being a transitional marker for G2/M [31]. SMP30 participates in the process of cellular senescence [32] affecting the proliferative process [33].

**Fig 4:**
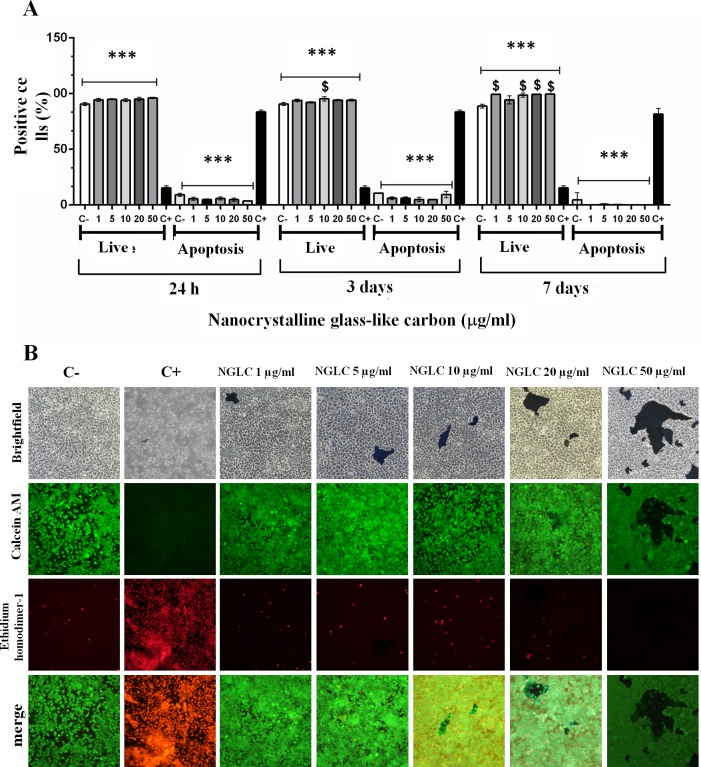
Cytotoxicity measurement via an apoptosis/necrosis assay. Values indicates apoptotic/necrotic cells expressed as percentage of positive-stained cells counted by flow cytometry after 24 hours, 3 days and 7 days of culture with nanocrystalline glass-like carbon (NGLC) microflakes at five different concentrations (1, 5, 10, 20 and 50 μg/ml) (**A**). Bright field (upper row) and immunofluorescence images (rows below) (**B**) showing the apoptosis/necrosis assay with SN4741 cells cultured in medium containing five different concentrations (1, 5, 10, 20 and 50 μg/ml) of NGLC microflakes. Calcein AM was used as vital immunofluorescence marker and EthD-1 as apoptosis marker, after 7 days of culture, studied also by merge images. For both experiments a positive control (C+) representing the use of 10% Triton X-100 in the culture was used to induce apoptotic processes, and negative control (C-) represents the culture without 10% Triton X-100 nor NGLC. The data are expressed as the mean± standard error of the means (SEM, n = 6). One-way ANOVA with Fisher’s post hoc test was used for comparison between groups; ***p<0.001 groups vs positive control (C+ = 10% Triton X-100), ^$^ p<0.01 groups vs negative control (C- = without 10% Triton X-100 nor NGLC).

Analysis of PH3 in control cells vs cells treated with 50 μg/ml NGLC after 24 hours and 7 days of culture showed a significant (p <0.05) decrease of the PH3 expression, i.e. at 24 h the cells are not active for cell division processes as is evidenced by an increase of SMP30. Subsequently, the analysis after day 7^th^ showed a much higher metabolic capacity respect to the control cells which is related to the increase in relative PH3 expression (Fig 3C). Cytotoxicity was analyzed using an apoptotic/necrosis assay as measured by flow cytometry (Fig 4A).

The test did not reveal significant differences between number of apoptotic cells (3–7%) with NGLC treatment and negative controls (C^-^ = samples without 10% Triton X-100 or NGLC), whereas a significant difference was found (p<0.001) respect to positive controls (C^+^ = samples with 10% Triton X-100 as apoptotic inducer), which reached 90±4% of apoptotic cells at the three culture conditions of 24h, 3 days and 7 days. In the same analysis, the number of live cells at 24 h showed no differences between the groups and the negative control but showed a significant (p<0.001) decrease compared to the positive control (13±2%). At 3 days of culture, we found only a significant change (p<0.01; 99±5% live cells) in one group (10 μg/ml) of NGLC treated cells. After 7 days, the live cell groups cultured with 1, 10, 20 and 50 μg/ml showed (100±10%) a significant (p<0.01) difference compared to the positive control cells (13±2%). The results described above corroborate the microscopic study (Fig 4B) after 7 days of culture by which, using bright field and immunofluorescence, we observed the markers Calcein AM as a vital marker and EthD-1 as apoptosis marker. It was possible to verify differences between the treatments groups and C^-^ and C^+^ in the Calcein AM images and the low presence of apoptotic cells observed in groups C^-^ and C^+^, which is also shown in the merge images. This demonstrates that NGLC does not promote apoptosis or necrosis processes as is evidenced by the EthD-1 immunofluorescent analysis (Fig 4A and 4B). No significant differences were observed at 24 hours of treatment at any NGLC concentration, not even at 50 μg /ml. At day 7 a significant increase in viability measured by Calcein AM from NGLC treated cells (including cells treated with a high dose 50 μg/ml) is observed. These data are also corroborated also by the MTT analysis (see Fig 3).

### Cell maturation

**Fig 5:**
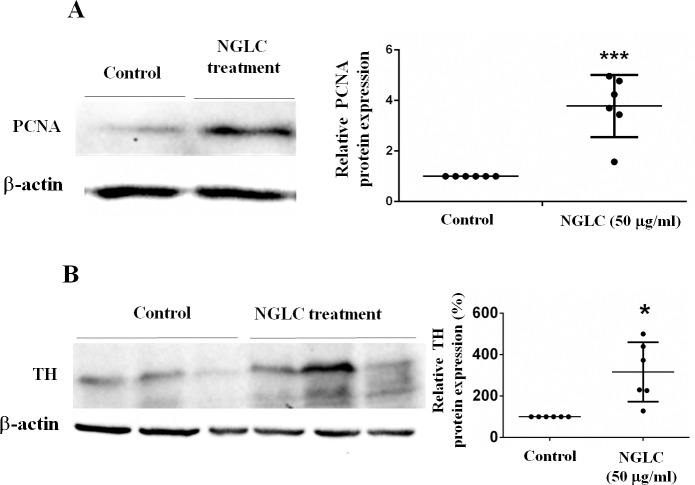
Proliferating cell nuclear antigen (PCNA) and thyroxin hydroxylase (TH) western-blots analysis. SN4741 cells were cultured for 7 days with a high concentration (50 μg/ml) of nanocrystalline glass-like carbon (NGLC) microflakes. PCNA (**A**) and TH (**B**) analysis for relative protein expressions were expressed as percentage. The data are shown as mean± standard error of the means (SEM, n = 3) and Student’s t tests were used for statistical significance between two groups; *p<0.05 and ***p<0.001.

PCNA western-blot (Fig 5A) after 7 days of culture with the highest NGLC concentration (50 μg/ml) demonstrated a significant (p<0.001) increase (200±48%) in protein respect to the housekeeping control (β-actin). The presence of NGLC microflakes (50 μg/ml) in the culture medium increased the ability to form networks using the microflakes as anchorage points (see Fig 2) representing this phenomenon a proof of induction of cellular maturation in culture.

For this reason we also examined whether the SN4741 cells after 7 days of culture with a high NGLC concentration (50 μg/ml) remained as precursor cells or matured into dopaminergic neurons. Most SN4741 cells became tyrosine hydroxylase (TH)-positive (Fig 5B) indicating the acquisition of a matured dopaminergic phenotype, as the western blot evidenced by a significant increase in TH (4 times) respect to control (Fig 5B).

### NGLC films evaluated as scaffolds

#### Analysis of cell morphology and cell distribution cultured on NGLC films

**Fig 6:**
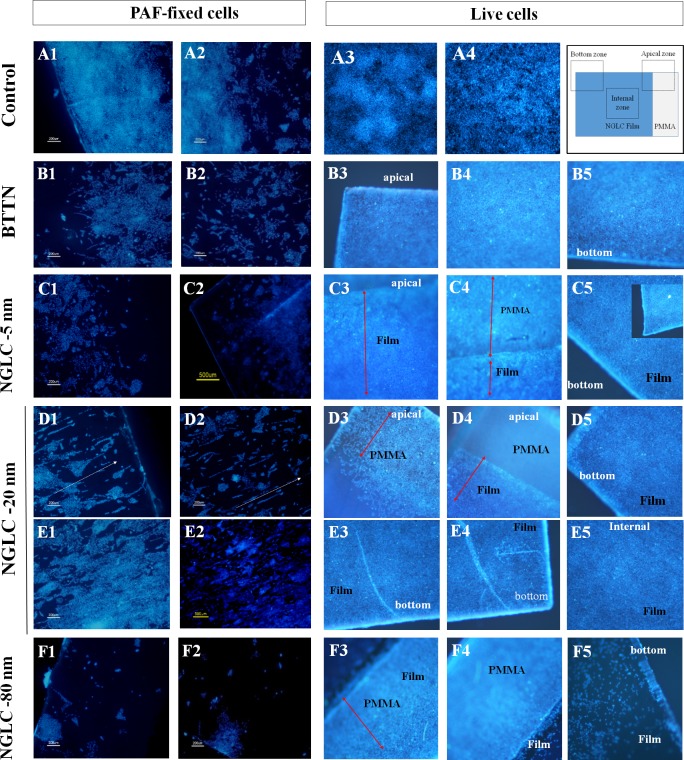
Influence of the NGLC film thickness in the SN4741 cell culture on the films. The two left pictures columns of the panel (PAF-fixed cells) show the microscopy images for DAPI immunofluorescence (nuclei maker: blue label) at low magnification (5X) showing different patterns of growth after 2 weeks. The three right pictures columns (live cells) show images obtained in vivo at low magnification (5 X) after labeling the nuclei with Hoechst 33342 (blue-marker). The diagram to the right in the first row shows the photographed areas and the limits between the NGLC film and the PMMA substrate. In the Apical Zone it is shown film and PMMA limits; Bottom Zone shows the end of the film area and in the Internal Zone the view is exclusively film surface. Fixed cells grown in monolayer on a glass slide (**A1-A2**), in plastic surface of the well (**A3-A4**), or in a baseline thermal-treated non-carbon (BTTN) film (**B1-B5**; Fig **B3**: apical zone, **B4**: internal zone, **B5**: bottom zone) as experimental controls. NGLC 5-nm-thick film (**C1-C5**), NGLC 20-nm-thick film (**D1-D5** and **E1-E5**) and NGLC 80-nm-thick film (**F1-F5**) were used for cell culturing on top of them. Areas corresponding to PMMA and NGLC films are differentiated in the pictures (**C3-C5**). Also spontaneously cell linear arrangement together with directionality of cell growth and cells orientation (**D1;** arrows) is shown in the bottom area of the film meanwhile growing in the internal film zone has the same cell arrangement effect (**D2 and E1-E2**). Separation between PMMA and the Film (**D4**) and the internal zone of the film (**D5**) are shown demonstrating how the cells firmly adhere to the film. Linear cell growing is observed in the bottom zone of the film (**E3-E4**) meanwhile the internal zone of the film shows adhered cells (**E5**). Fixed cells grown on top of the NGLC 80-nm-thick film (**F1-F2**) showing the limits of PMMA substrate (**F3-F4** arrow) and film and a detailed film bottom is also shown (**F5**).

The ability of NGLC films as scaffold for cellular nesting or adhesion is demonstrated in different cell cultures (Fig 6), where images of DAPI immunofluorescence (blue label) at low magnification (5X) (Fig 6, two columns on the left) showed different growth patterns after 7 days of culture after a seeding of around 10000 cells per NGLC and baseline thermal-treated non-carbon (BTTN) films or glass slide (as control). We also demonstrate the cell adhesion to the films in vivo using Hoechst 33342 staining as nuclear marker (Fig 6, three columns on the right) although the high cell density, around 200000 cells per film or plastic surface (control) gives no information about the effect on the distribution of SN4741 cells. Furthermore, we found that cultures on 20 nm-thick-films showed a spontaneous directional and linear distribution (Fig 6D1 and 6D2 -arrows- and Fig 6E1 and 6E2). This particular distribution does not seem to be due to a structural artifact of the film itself (see S1 Fig), but to other factors not determined in this work. Even in internal zones of the film with high cell density it is verified the fact of such special cells distribution (Fig 6E1 and 6E2). The directionality is not observed in the negative controls (Fig 6A1 and 6A2) neither in the controls grown on plastic supports (Fig 6A3 and 6A4) nor on BTTN (Fig 6B1–6B5). The 80nm film (Fig 6B1–6B5) seems to be the one with the lowest number of cells maybe due to its higher carbon content. Nevertheless, cells cultured at long term (2 weeks) and fixed showed low number of cells (S2 Fig), however the NGLC 20 nm-thick-film has a larger number of adhered cells when compared to those of the 5 and 80 nm films.

#### Effect on cell maturation

**Fig 7:**
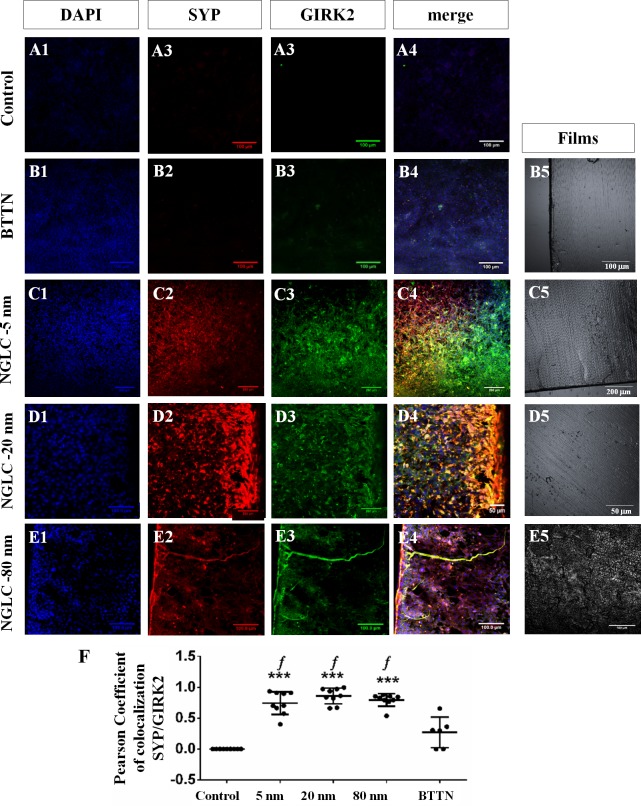
Confocal immunofluorescence of functional and cell maturation markers in the SN4741 cells at long term in culture. Nuclear marker DAPI (blue-label), synaptophysin (SYP: red-label) and G-protein-regulated inward-rectifier potassium channel 2 (GIRK2: green-label) proteins immunofluorescence were localized in SN4741 cells cultured on plastic wells (**A1-A4**), baseline thermal-treated non-carbon (BTTN) film (**B1-B4**), NGLC 5-nm-thick film (**C1-C4**), NGLC 20-nm-thick film (**D1-D4)** and NGLC 80-nm-thick film (**E1-E4**). Colocalization (merge) as yellow-label and the specific protein markers related with functional differentiation is also quantifying (**F**). Brightfields images of BTTN (**B5**) and 5 nm (**C5**), 20nm (**D5**), 80nm (**E5**) NGLC films are located in the right column of the panel. Colocalization percentage of specific protein markers for functional differentiation SYP and GIRK2 was also calculated (**F**) by means of Pearson's correlation coefficient, as previously described by Dunn et al. (2011) using the Fiji-ImageJ software and the plugin ‘Colocalization Threshold’. Data are expressed as mean±standard deviation (SD, n = 6); **p<0.01 and ***p<0.001 vs control (cells cultured in plastic wells); ‘*f*’ = p<0,001 mark differences between NGLC samples and baseline thermal-treated non-carbon (BTTN) film.

When the SN4741 cells were cultured during 4 weeks on top of slides as control (Fig 7A1–7A4), or on BTTN support (Fig 7B1–7B4) as control of the carbon film, or on top of the 5-nm-thick (Fig 7C1–7C4), 20-nm-thick (Fig 7D1–7D4) and 80-nm-thick NGLC films (Fig 7D1–7D4), we observed a differential behavioral pattern thickness dependent, as demonstrated by immunofluorescence of the cell differentiation and the cellular communication markers SYP and GIRK2.

The immunofluorescence results were quantified (Fig 7F) to establish the degree of SYP/GIRK2 markers colocalization in cells cultured on NGLC films with different thicknesses by using Pearson's correlation coefficient [26] where the maximum 1 represents the 100% of cells colocalizing both antibodies being 0 as no colocalization at all. The three types of NGLC films with different thicknesses showed elevated significant (p<0.001) differences versus control (cells cultured without NGLC) for NGLC 5-nm-thick film (Pearson’s coefficient = 0.7428±0.1843; n = 9) and 20-nm-thick film (Pearson’s coefficient = 0.8578±0.1260; n = 9) and lower significant differences (p<0.01) for 80-nm-thick film (Pearson’s coefficient = 0.7928±0.1030 n = 9) when compared to the control (Person’s coefficient = 0±0; n = 9) or BTTN (Person’s coefficient = 0.2690±0.2472; n = 6) (Data expressed as mean±SD).

**Fig 8:**
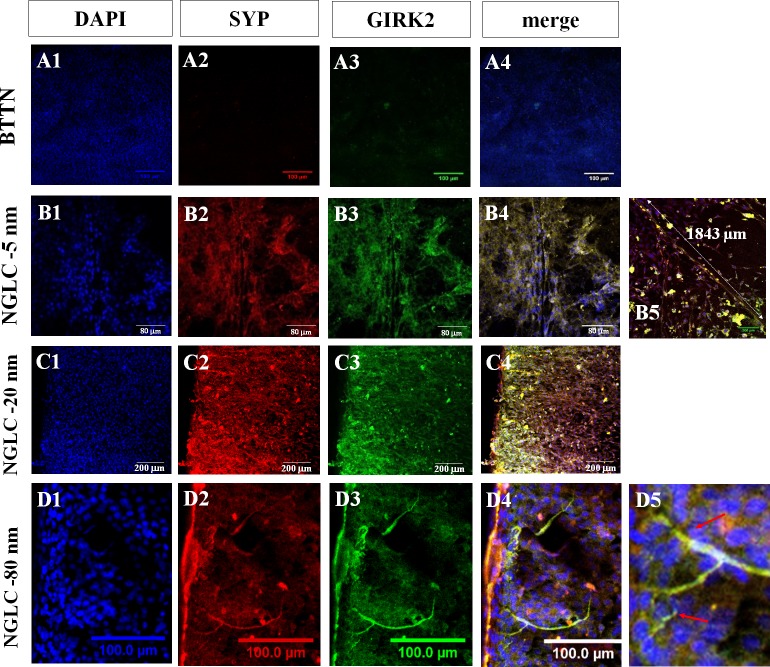
Neural-like processes induced by NGLC films. Confocal immunofluorescence microscopy images showing morphological and structural effects of NGLC films on the SN4741 cells cultured on top of them. Nuclear marker DAPI (blue-label), synaptophysin (SYP: red-label) and G-protein-regulated inward-rectifier potassium channel 2 (GIRK2: green-label) proteins are shown as well as their colocalization (merge: yellow-label). The positive DAPI staining (**A1, B1, C1, D1**) showed a monolayer culture image without visible sign of differentiation. Cells cultured on BTTN films presented poor signal for SYP (**A2**) and GIRK2 (**A3**) markers. Cultures on NGLC 5-nm-thick films showed increasing neural-like processes with SYP/GIRK2 colocalization (**B1-B4**) where an axon-like process is measured (1843 μm) (**B5**). These processes were never seen in control cultures neither in BTTN cultures. For the measurement of the axon length Image J/Fiji software with the plugin ‘Microscope measurement Tools’ were used. It was observed with NGLC 20-nm-thick films (**C1-C5**) a greater synaptic capacity (SYP/GIRK2 signal) where a cell polarized and oriented growing towards the limit of the NGLC film (**C4**) (see also Fig 7, D4). Positive SYP highly expressed is a proof of a functional characteristic of the cells to address their processes to the outer limit. In the cell culture on NGLC 80-nm-thick films (**D1-D5**) axon-like processes (D3), arborization (D4) and denditric-like formation (D5) can be observed due to the cellular development and maturation and not to any artifactual folding of the film.

Although GIRK2 was found to be present in prominent axon-like images (Fig 8) the cells on top of the highest thickness film (80-nm) possessed the lowest (70%) SYP coexistence rate (see Fig 7F).

Furthermore, NGLC films present architectural characteristics revealing the capacity to configure cell networks (Fig 8B1–8B4) after 4 weeks in culture. The merged image (Fig 8B5) from the cells cultured on the NGLC 5-nm-thick film showed dendrite-like or axon-like processes that were positive for both SYP and GIRK2 proteins showing some of them with a length (1.83 mm) never observed in controls or BTTN samples. We also observed neurite-like processes that were positive for GIRK2 in cells cultured on top of the 80-nm-thick film (Fig 8D1–8D4). This reaction demonstrate that the process is not an artifact either (Fig 8D5) or a film folding which is not possible due to the film structure (S1 Fig) while an specific signal for both SYP and GIRK2 is clearly distinguished (Fig 8D2 and 8D3 and S3 Fig)

## Discussion

Glass-like carbons are a class of disordered sp2-hybridized carbon, whose microstructure consists of randomly oriented fullerene-like fragments of different sizes. This amorphous microstructure reduces their electrical and thermal properties as compared to highly crystalline carbons as graphene, but grants other interesting properties such as high temperature stability, extreme resistance to chemical corrosion, or high hardness [14]. In fact, these properties provided by glass-like carbons are shown to be suitable for chemical monitoring in vivo [34][35]. On the other hand, the production of amorphous carbon thin films offers high scalability due to its easy-to-replicate microstructure in comparison to highly crystalline structures. In this regard, both electrical and optical properties can easily be tuned by controlling the gas composition during the CVD [15]. Other carbon-based thin films composites showing similar electrical and optical properties can be produced by dispersing graphene oxide powders [36], but do not provide the physical continuity of a single and continuous thin film.

In this work, we have studied for the first time the biocompatibility and cell maturating influence of a particular NGLC by using the SN4741 cell line from substantia nigra DA cells derived from transgenic mouse embryos and cultured with either increasing concentrations of NGLC microflakes or on top of NGLC films with different thicknesses. Culturing the cells in medium with increasing concentrations of NGLC microflakes allowed us to observe dose-dependent effects on the NGLC capacity to promote cell networking and thus neuronal capability and metabolism.

One of the effects observed in cultures with NGLC microflakes is the capacity to provide cells anchorage points from which dose-dependent networks are organized. This special behavior may be related to the assertion that surface topography and physicochemical properties determine cell adhesion and proliferation [37][38]. In this regard, our amorphous carbon composed of nano-crystalline clusters (as detected by TEM and Raman analysis) provide a surface with high density of defects which may play a role as anchorage points improving the cell adhesion and proliferation. Similarly, the surface topography of our carbon film samples transferred on PMMA, AFM studies demonstrated increasing roughness, especially in those samples of carbon films with 20 and 80 nm thicknesses. In particular, the 20-nm-thick film provide a planar surface with peak-to-valley values up to 40 nm meanwhile a more rugged surface is found on 80-nm-thick samples, with peak-to-valley values from 150 nm-600 nm. Concerning the adhesion and proliferation results, smooth wrinkles provided by 20-nm-thick films appear to be more relevant. In fact, these properties could be related to the spontaneous networking capability which plays a decisive role to test NGLC capacity to induce cellular connectivity among SN4741 neural cells.

Other carbon materials as nanodiamonds [13] have been frequently used as micropatterned substrates capable to induce neuronal cell growth though the authors obtained a low cell concentration and no spatial organization nor architectural changes in the culture in contrast to NGLC films. This suggests that the benefit of nanodiamonts as an optimum material to favor neuronal functionality is questionable in contrast to the induced spontaneous ability to establish communication pathways of our no patterned NGLC material.

Considering the possibility that NGLC could be used in vivo as an inducing material for regenerative therapies, the inherent ability of NGLC to stimulate the structural organization of nerve tissue is a fact that must be considered for application. In contrast, other authors [13][39] who use nanomaterials with an established pattering draw away from the existence of a true neural networks and connectivity which exists within any nerve tissue [40].

Notably, our results on cell metabolism reveal that there is a process of early cellular adaptation in the first 24 hours of NGLC treatment, where a significant decrease in cellular metabolism compared to the control is observed at a high concentration (50 μg/ml). This decrease was not associated with an increase in cell death (confirmed by EthD-1 analysis). On the contrary, the levels of vital activity measured by Calcein AM are equal both in the samples with or without NGLC treatment. These findings on biocompatibility, in addition of being the first described for this particular carbon nanomaterial, are agree in part with other studies of carbon-based nanoparticles [4][19][20]. In contrast, the decrease in metabolic activity using the same concentration of NGLC microflakes was associated with a decrease in PH3 that could be delaying the processes of cell division and consequently lowering the synthetic capacity of the metabolic MTT transformation. These results suggest that prior to the process of dopaminergic differentiation; SN4741 cells enter a process without cell division, which is necessary for cell transformation into a neuronal maturation process. This fact has been corroborated by the increase of the senescence marker SMP30 as it was evidenced in our results at 24 h in cells treated with NGLC. After 7 days of culture, a metabolic increase is recorded along with a tendency to increase the PH3expression, i.e. the cells are actively dividing in addition to possessing a significantly high replicative activity as it is evidenced by PCNA analysis [41]. This replicative and proliferative processes, suppose a greater energetic cost for the cell and coincides with an increase of cellular metabolism and Calcein AM activity. Although time responses have been carried out in graphene studies [42] it was used heterogeneous cell cultures, where cells are metabolically more active than others, and this fact may be hiding the true action on MTT. Since SN4741 cells are clonogenic, we performed experiments to synchronize the cell growth of SN4741 cells prior to start the present study [43], so that it allows us to determine changes in cell cycle. The result suggests that the time the cells should be in contact with NGLC is determinant considering cell therapy possibilities. As it is shown in our result suggests that the ratio of proliferation vs metabolism may be consider for choosing the best time for cellular implantation into a certain tissue as expected time to obtain successful tissue capability development. This aspect may depend on the effect of the nanomaterial on the cell in terms of neural capacity, as well as on the capacity of the cell to be able to orient properly, develop and differentiate on the tissue.

Focusing our work on cell replacement therapies for damaged DA cells in Parkinson’s disease [17] or considering any of the multiple possibilities for neural cell growth, these NGLC films could be considered to produce patches for cell regeneration. Furthermore, we have observed, using our NGLC 5-nm-tick-films, spontaneous neurite- and axon-like sprouts reaching several mm elongation. Notably, this result reproduced in SN4741 cells suggests that NGLC might be an interesting material to study the ability to rebuild among others nervous system tracts in relation to the Parkinson’s disease by favoring axonal sprout projecting onto the tracts linking the areas affected by the disease [44]. In addition, NGLC 20-nm-thick-film showed a clear capacity to induce spontaneous neuronal orientation and a very high degree of neuronal polarization. The importance of this phenomenon has been successfully reproduced in a 3D neuronal networks [45] or through a pattering model [39] which induced a directional neuronal growing but lucks of a true functional communication between neurons. The NGLC effects on cell orientation confirmed by the SYP signal (outwardly oriented expression) observed in the 20-nm-tick-films at long term in culture pointed out this particular carbon film as a candidate for studies in PD regenerative studies. It has also been described using induced pluripotent cells and collagen scaffolds the importance of neurite elongation and the position of the soma to mimic the layered brain structure [45].

When the cells grow on top of the NGLC films, cellular responses were assessed, and the best result on maturation that was obtained after 7 days of culture. Good adhesion was also observed, although the surfaces of the films were hydrophobic to a certain extent. Because we irradiated shortly the film with UL at 185 nm before use for culture to avoid microplasma, it can be assumed that this practice provides a certain degree of reversible wettability hydrophobic to hydrophilic transition that renders the surface of NGLC highly biocompatible and facilitates adhesion, as it has been recently published with the same glass-like carbon [15]. Nevertheless, our results show that this nanomaterial, although somewhat hydrophobic (NGLC 80-nm-tick film with the higher carbon concentration), facilitates viability, neuronal functionality, differentiation and cell communication. Furthermore, it is known that controlled surface morphology (topography/roughness) and hydrophilicity are considered important for cell differentiation [5] in a range of biomedical uses, such as the creation of nano-biointerfaces for molecular medicine [7]. The reason why differentiation and neurite-like outgrowth are stimulated by using NGLF films are not yet determined. What is relevant is that this phenomenon is not related to any growth factor added to the culture medium nor to the coating medium, as is done in other works [13] who use a medium called ‘attachment medium’ composed by laminin and poly-L-orthinine combined with nanodiamons. Since, it is known that laminin is a potent neuroinductor and neurodifferentiator [46] the neurite extension could be consequently a phenomenon induced by laminin itself. However, it is very relevant that NGLC is not combined with any coating with a differentiating and guiding effect.

Regarding the surface topography of our carbon film samples transferred on PMMA, AFM studies demonstrated increasing micro roughness as far as NGLC film thicknesses increases. Therefore, these smooth wrinkles appear to be relevant to the surface influence. However the roughness is not an artifact that affects the axon sprouting as we demonstrated in our results but the presence of micro roughness may be a determinant factor to facilitate adhesion points allowing cellular anchorage, and therefore adhesion and axonal outgrowth as it has been previously reported [47].

However, we speculate that the thickness of the films could be of crucial importance for improving the degree of cell maturation and differentiation, as we observed in the cell cultures on the 20- or 80-nm-thick NGLC films. The results obtained for SYP and GIRK2 markers and by studying their coexistence evidenced a much higher capacity of the 20-nm-thick films to provide better functional neuronal-like labeling than that observed with the 5-nm-thick and 80-nm-thick films, and consequently if the post-synaptic communication exists, the nervous stimulus could exist. However, the surface exposed to the cell culture is theoretically the same in all three (5-, 20- or 80-nm-thick) NGLC films used in our experiments, but not the back side of the films, which is primarily carbon and which yields the thickness of the films [15]. Thus, the thickness of the films can influence not only the rigidity of the films but also their optical and electrical characteristics, and because the 20-nm-thick films are twice as conductive for electricity as the 5-nm-thick films while maintaining certain degree of transparency (which is not provided by the opaque 80-nm-thick) [15], we may conclude that the neural capability together with transparency of the NGLC films are a clue for the degree of cell maturation that seems to be directly related to the specific accumulation of the carbon molecules composing the film.

## Conclusions

This particular nanometer-thin NGLC is biocompatible for SN4742 cells. NGLC induces cell differentiation and stimulates neuronal functional capabilities. We observed a direct relationship between the thickness and roughness of the films and cell maturation, for which the 20-nm-thick films were the best for inducing orientation, number of adhered cells and cell maturation. Because this nanomaterial is synthesized under highly controlled parameters, it could offer a powerful and reproducible platform for use in biomedical applications, such as for use in neural tissue engineering and in the development of biocompatible devices.

## Supporting information

S1 Fig Microscopic imagines of the films. Baseline thermal-treated non-carbon-film (BTTN) and different films of nanometer-thin nanocrystalline glass-like carbon film (NGLC): 5 nm, 20 nm and 80 nm used in the cell culture experiments.
(TIF)

S2 Fig Quantification of total number of cell by nuclei counting. It is shown the total number of DAPI positive cells in PAF-fixed samples (**A**) of cell culture at long term on three different samples of control (plastic wells), baseline thermal-treated non-carbon-film (BTTN) and NGLC film randomly chosen. The quantification of live cells (**B**) was also performed by means of Hoechst 33342 staining. ImageJ/Fiji plugin Cell Counter was used to quantify nuclei.
(TIF)

S3 Fig Confocal images of cells at long term. Cell cultured on NGLC 20 nm film (**A**) using Confocal Regional Analysis tool. The figure shows how the cells can adhere along the film surface (Bar 2000 μm). It is shown an enlarged area (**B**) in which it is found high synaptophysin (SYP: red-label) expression. A small area from Fig A, is also enlarged showing SYP, G-protein-regulated inward-rectifier potassium channel 2 (GIRK2: green-label) and DAPI stains as well as the merge image. These picture panels demonstrate that the possible artifacts of the film did not affect the neural-like processes described in Fig 8.
(TIF)
