# Supplementary figures and images for: Cell survival and differentiation with nanocrystalline glass-like carbon using substantia nigra dopaminergic cells derived from transgenic mouse embryos
Source: PLoS One. 2017 Mar 23;12(3):e0173978. doi: 10.1371/journal.pone.0173978 (PMC5363826; doi:10.1371/journal.pone.0173978)

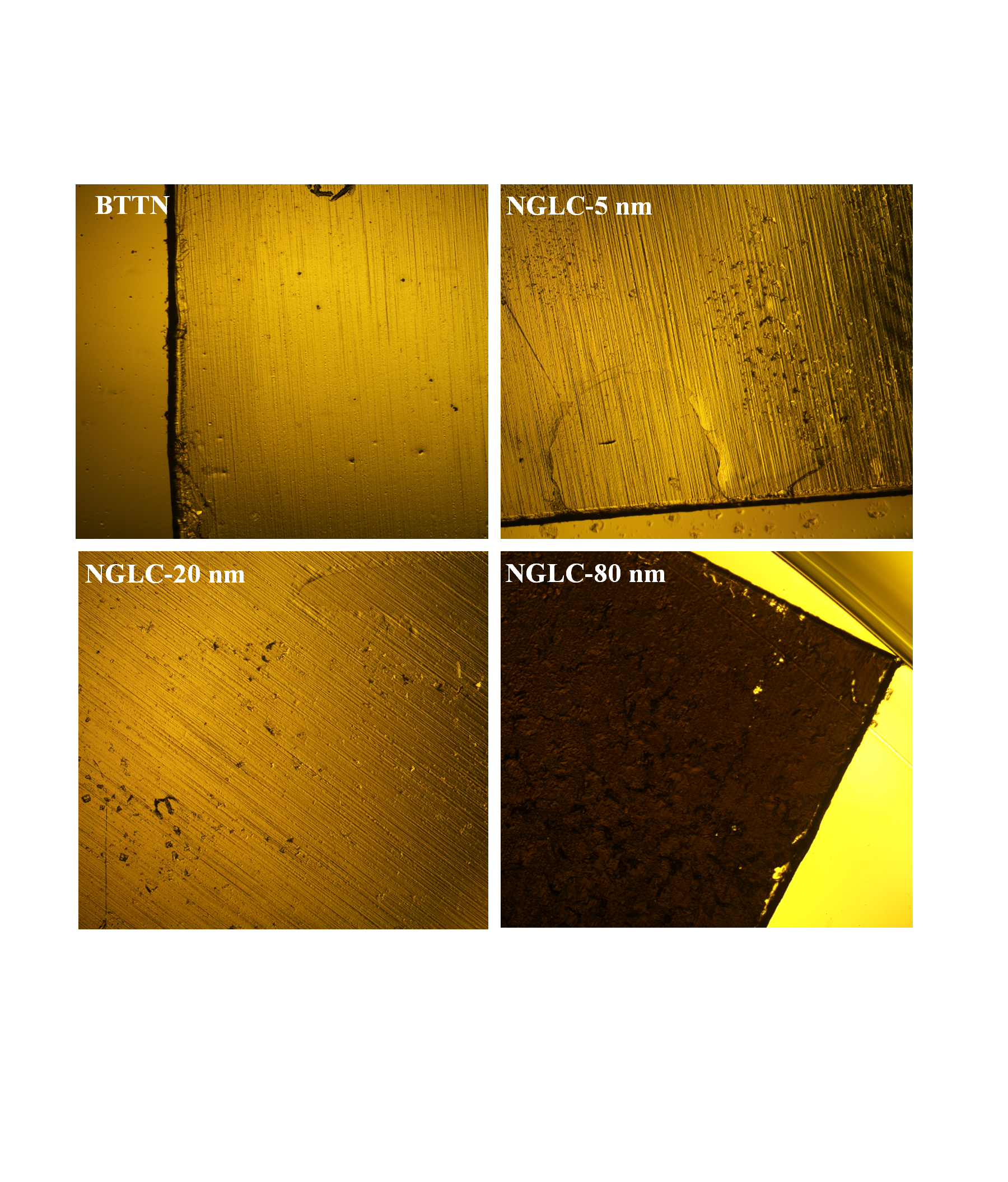

Supplement: S1 Fig — Baseline thermal-treated non-carbon-film (BTTN) and different films of nanometer-thin nanocrystalline glass-like carbon film (NGLC): 5 nm, 20 nm and 80 nm used in the cell culture experiments. (TIF) [file pone.0173978.s001.tif]

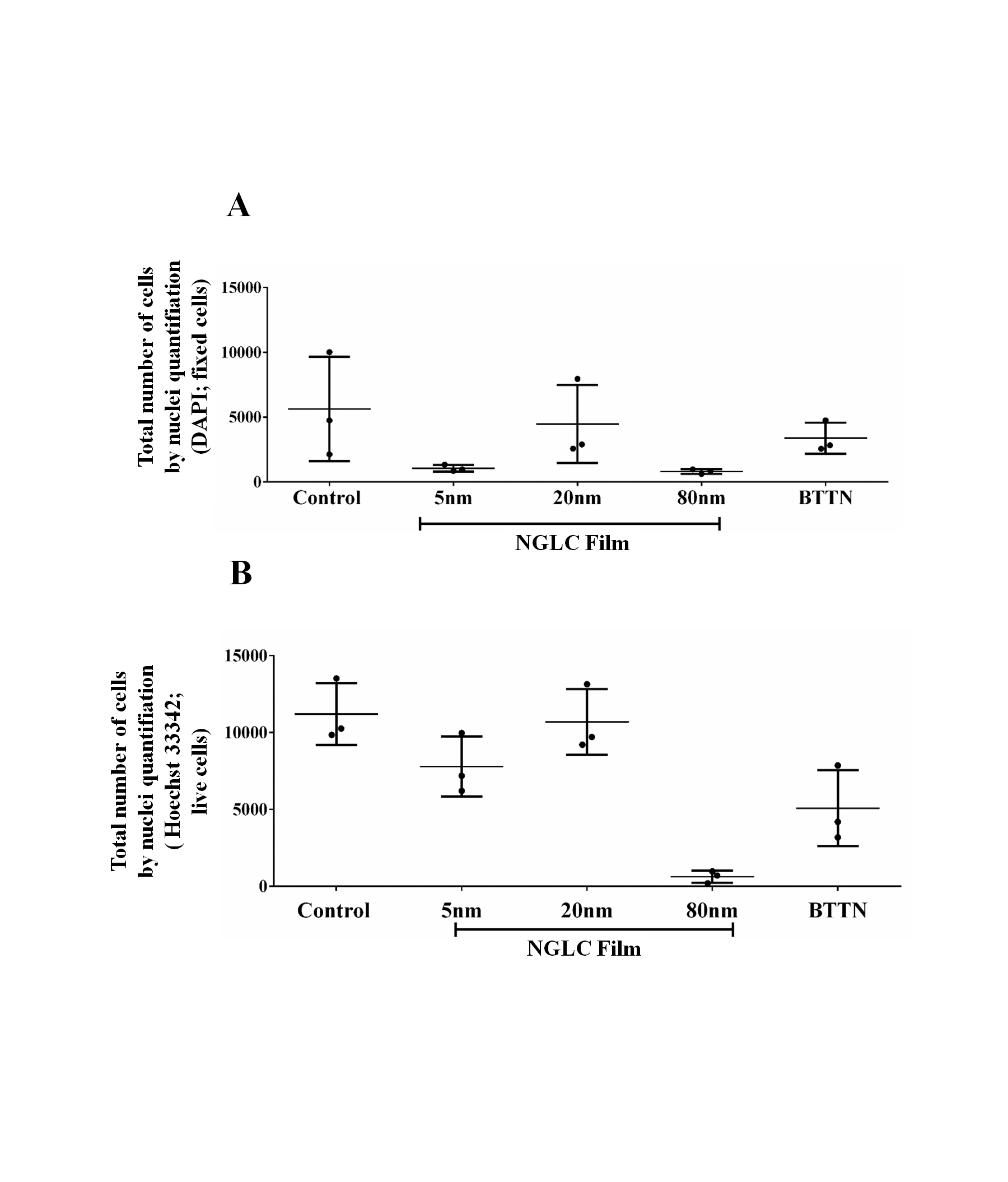

Supplement: S2 Fig — It is shown the total number of DAPI positive cells in PAF-fixed samples (A) of cell culture at long term on three different samples of control (plastic wells), baseline thermal-treated non-carbon-film (BTTN) and NGLC film randomly chosen. The quantification of live cells (B) was also performed by means of Hoechst 33342 staining. ImageJ/Fiji plugin Cell Counter was used to quantify nuclei. (TIF) [file pone.0173978.s002.tif]

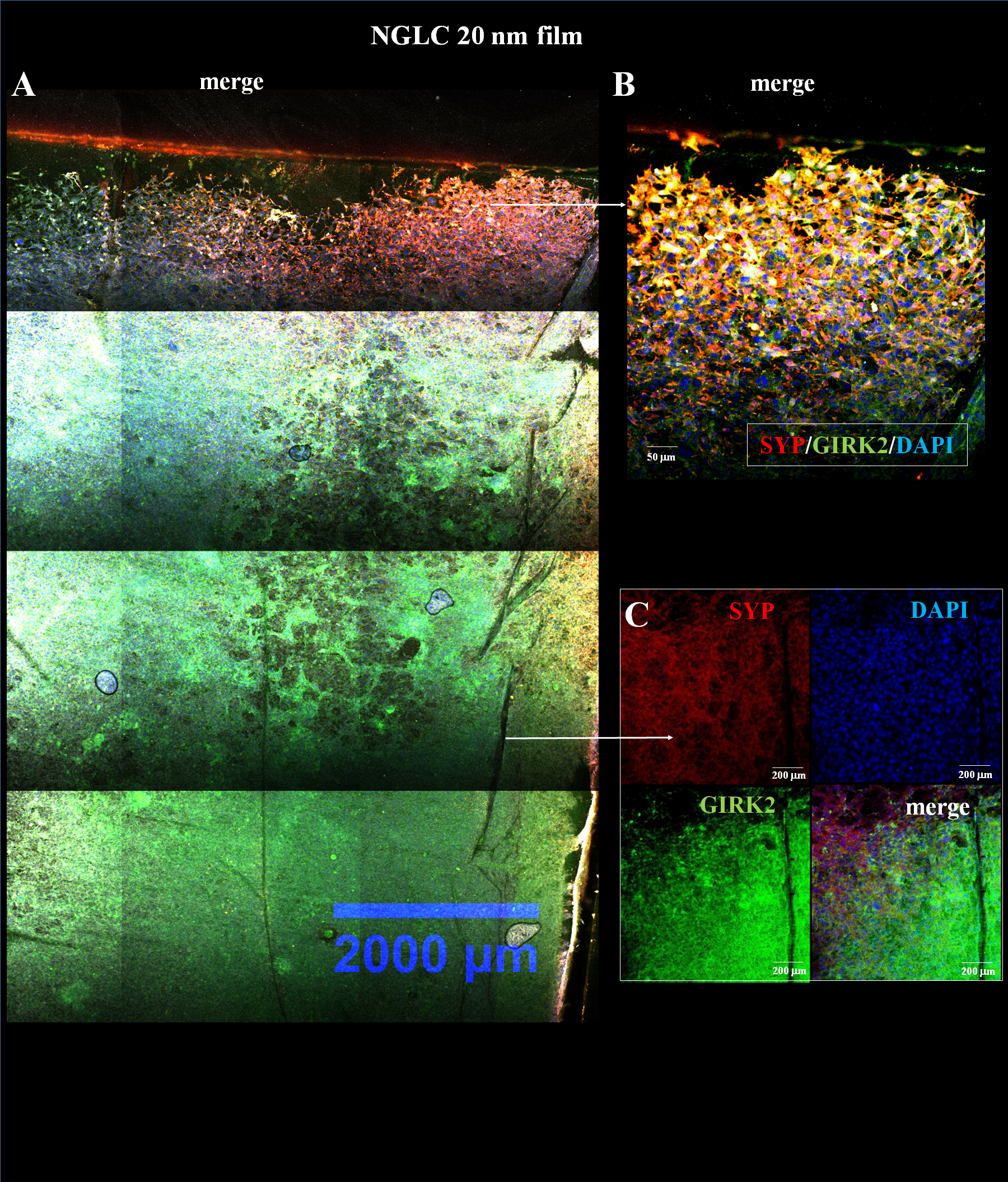

Supplement: S3 Fig — Cell cultured on NGLC 20 nm film (A) using Confocal Regional Analysis tool. The figure shows how the cells can adhere along the film surface (Bar 2000 μm). It is shown an enlarged area (B) in which it is found high synaptophysin (SYP: red-label) expression. A small area from Fig A, is also enlarged showing SYP, G-protein-regulated inward-rectifier potassium channel 2 (GIRK2: green-label) and DAPI stains as well as the merge image. These picture panels demonstrate that the possible artifacts of the film did not affect the neural-like processes described in Fig 8. (TIF) [file pone.0173978.s003.tif]
